# Dietary patterns and chronic kidney disease: a cross-sectional association in the Irish Nun Eye Study

**DOI:** 10.1038/s41598-018-25067-7

**Published:** 2018-04-27

**Authors:** Euan N. Paterson, Charlotte E. Neville, Giuliana Silvestri, Shannon Montgomery, Evelyn Moore, Vittorio Silvestri, Christopher R. Cardwell, Tom J. MacGillivray, Alexander P. Maxwell, Jayne V. Woodside, Gareth J. McKay

**Affiliations:** 1 Centre for Public Health, Queen’s University Belfast, Belfast, Northern Ireland; 2 Department of Ophthalmology, Belfast Health and Social Care Trust, Royal Hospital, Belfast, Northern Ireland; 3 Centre for Clinical Brain Sciences, The University of Edinburgh, Edinburgh, Scotland; 4 UKCRC Centre of Excellence for Public Health, School of Medicine, Dentistry and Biomedical Sciences, Queen’s University Belfast, Belfast, BT12 6BJ United Kingdom

## Abstract

Associations between dietary patterns and chronic kidney disease are not well established, especially in European populations. We conducted a cross-sectional study of 1033 older Irish women (age range 56–100 years) with a restricted lifestyle. Dietary intake was assessed using a food frequency questionnaire. Renal function was determined by estimated glomerular filtration rate. Two dietary patterns were identified within the study population using factor analysis. A significant negative association was found between unhealthy dietary pattern adherence and renal function in both unadjusted and adjusted models controlling for potential confounding variables (p for trend <0.001), with a mean difference in estimated glomerular filtration rate of −6 ml/min/1.73 m^2^ between those in the highest fifth of adherence to the unhealthy dietary pattern compared to the lowest, in the fully adjusted model. Chronic kidney disease risk was significantly greater for the highest fifth, compared to the lowest fifth of unhealthy dietary pattern adherence in adjusted models (adjusted odds ratio = 2.62, p < 0.001). Adherence to the healthy dietary pattern was not associated with renal function or chronic kidney disease in adjusted models. In this cohort, an unhealthy dietary pattern was associated with lower renal function and greater prevalence of chronic kidney disease.

## Introduction

Diet and nutritional intake are associated with important risk factors for chronic kidney disease (CKD) including type 2 diabetes^1^ and hypertension^2^, yet dietary guidelines for prevention of CKD are lacking due to a dearth of evidence. To better understand healthy dietary intake in target populations, a dietary pattern based approach may prove more informative than studies of individual nutrients as dietary patterns consider the complex milieu of consumed nutrients and account for synergistic and competitive interactions between dietary components of both known, and as yet unknown, mechanistic action^3^. Indeed, recent guidelines for the prevention and management of CKD-related conditions such as cardiovascular disease and diabetes have focused on dietary pattern based recommendations^3,4^.

Associations between dietary patterns and CKD have been investigated previously. Most studies to date have focused on *a-priori* derived dietary patterns, a score based approach which indicates adherence to a particular predefined diet. Using this *a-priori* based approach, the DASH (Dietary Approaches to Stop Hypertension) and Mediterranean diet have both been studied in the context of CKD^5–11^. A DASH-style diet has previously been reported to lower the risk of cardiovascular disease^12^, type 2 diabetes^1^, stroke^13^, and cancer^14^, and reduce the risk of kidney stone formation^15^. Consumption of a Mediterranean style diet has also been associated with lower risk of diabetes and cardiovascular disease^16^. Previous studies have reported associations between increased adherence to the DASH diet and reduced risk of estimated glomerular filtration rate (eGFR) decline^5^ and CKD^6,7,17^. Greater adherence to a Mediterranean style diet has also been associated with improved creatinine clearance^8^, reductions in CKD^9,10,18^, rate of eGFR decline^9^, and albuminuria^11^. Furthermore, a recent meta-analysis of dietary pattern studies has shown that among individuals with CKD, healthy dietary patterns are associated with lower mortality, but no reduction in risk of end stage renal disease^19^.

In contrast to the *a-priori* approach, an *a-posteriori* approach using factor analysis to identify existing dietary patterns within a study population can also be used. Such *a-posteriori* derived dietary patterns have been examined in relation to renal function and damage in a limited number of studies based in the US^5,20,21^ and China^22^, but there is a lack of evidence in European populations. Of those conducted in the US, the Nurses’ Health Study found that consumption of a “Western” dietary pattern was associated with albuminuria while a “Prudent” dietary pattern was not associated with albuminuria or reduced renal function^5^. Similarly, in the Multiethnic Study of Atherosclerosis, dietary patterns characterized by consumption of low-fat dairy, wholegrains, fruits and vegetables were associated with lower albumin/creatinine ratio^20^. In contrast, the Reasons for Geographic and Racial Differences in Stroke study identified a more diverse set of dietary patterns that reflected the South-Eastern US locality of the study^21^. They found no associations with end-stage renal disease but reported higher mortality rates in those with CKD who were consuming a “Southern” dietary pattern, characterized by greater intake of fried food, organ meats, and sweetened beverages, and lower rates in those consuming a “Plant-based” dietary pattern. Another study, based in China, noted that the associations between dietary patterns and CKD were heavily influenced by industrial pollutants, with conceptually healthy traditional diets being associated with increased CKD risk^22^, further highlighting the importance of locally relevant dietary pattern identification.

Population-specific dietary patterns have the advantage of cultural specificity and may facilitate a better understanding of the importance of specific foods/food groups present or absent in other healthy dietary patterns such as the Mediterranean and DASH diets. The aim of this study was to examine the association between *a-posteriori* dietary patterns and renal function identified within a highly specific cohort of older women with relatively stable and restricted lifestyle behaviours.

## Results

The mean age of participants was 76 years (standard deviation [SD]: 8; Table 1), and according to body mass index (BMI) classification 41% (n = 421) were of normal weight. Approximately 3% (n = 31) had diabetes, hypertension had been clinically diagnosed in 60% (n = 566) of participants, and 61% (n = 557) of participants had CKD stage 3–5 (eGFR <60 ml/min/1.73 m^2^).

**Table 1:** Sample mean values and frequencies, for total sample and by fifths of adherence to the Healthy Dietary Pattern.

Healthy Dietary Pattern	Sample n = 1033	Q1 n = 206	Q2 n = 208	Q3 n = 206	Q4 n = 207	Q5 n = 206	*P*
Age, years (SD)	76.3 (8.0)	79.1 (8.1)^a^	77.4 (7.9)^b^	76.3 (7.61)^b^	75.9 (7.8)^b^	73.1 (7.4)^c^	<0.001^†^
BMI, kg/m^2^ (SD)	24.6 (5.1)	23.7 (4.5)^a^	24.3 (5.6)^ab^	25.4 (5.3)^ab^	24.5 (5.1)^ab^	24.9 (4.8)^b^	0.03^†^
Hypertension, n (%)	566 (60.1)	108 (60.7)	117 (59.1)	104 (59.4)	126 (64.3)	111 (57.2)	0.69
Alcohol, n (%)	77 (8.2)	13 (7.3)	19 (9.6)	10 (5.7)	16 (8.2)	19 (9.8)	0.60
Ever smoked n (%)	35 (3.7)	4 (2.2)	9 (4.5)	7 (3.4)	6 (3.1)	9 (4.7)	0.70
IHD, n (%)	107 (11.4)	14 (7.9)	25 (12.6)	19 (10.9)	27 (13.8)	22 (11.4)	0.46
CVA, n (%)	30 (3.2)	7 (3.9)	8 (4.0)	6 (3.4)	7 (3.6)	2 (1)	0.43
Diabetes mellitus [any] n (%)	31 (3.3)	4 (2.2)	7 (3.5)	4 (2.3)	8 (4.1)	8 (4.1)	0.74
Type 2 diabetes,	28 (3)	2 (1.1)	6 (3)	4 (2.3)	8 (4.1)	8 (4.1)	0.39
eGFR <60 mL/min/1.73 m^2^ n (%)	557 (60.6)	114 (65.9)	109 (56.5)	112 (65.5)	117 (61.3)	105 (55.0)	0.10
eGFR <60 & ≥30 mL/min/1.73 m^2^ n (%)	534 (58.1)	108 (62.4)	105 (54.4)	107 (62.6)	113 (59.2)	101 (52.9)	0.20
eGFR <30 mL/min/1.73 m^2^ n (%)	23 (2.5)	6 (3.5)	4 (2.1)	5 (2.9)	4 (2.1)	4 (2.1)	0.88

SD, standard deviation; IHD, ischemic heart disease; CVA’ cerebrovascular accident; BMI, body mass index; eGFR, estimated glomerular filtration rate; Q1-5, fifths of dietary pattern adherence (Q1 lowest fifth, Q5 highest fifth). *Significant difference from lowest fifth in pairwise comparison for categorical variables via Mann-Whitney U test. ^†^P-value from ANOVA. ^a,b,c^Where ANOVA P-value is significant, groups which are not significantly different from each other (by Student-Newman-Keuls) have been given the same subscript whereas groups which are different have been given different subscripts.

Food frequency questionnaire (FFQ) data were obtained for 1033 participants. Principal component analysis identified two major dietary patterns that were labelled as ‘healthy’ (factor 1) and ‘unhealthy’ (factor 2) and which together accounted for 16% of the sample variance. These dietary patterns comprise foods for which intake is most closely correlated and constitute naturally occurring dietary patterns. The factor loadings for both dietary patterns are presented in Table 2. The ‘healthy’ dietary pattern was defined (in decreasing order of factor loadings) by lutein/zeaxanthin-rich vegetables, green leafy vegetables, alliums, vegetables, fruit, tomatoes, legumes, nuts, oily fish, low fat dairy products, pizza, dressings/sauces/condiments, wholegrain breakfast cereal and red meat. The ‘unhealthy’ dietary pattern was defined (in decreasing order of factor loadings) by crisps, chips, alcohol, high fat dairy products, soups, desserts, sugars and sweets, wholegrains, dressings/sauces/condiments, processed meat, potatoes, eggs, refined grains, refined breakfast cereal, chocolate, vegetables, red meat, white fish and shellfish.

**Table 2:** Factor loadings for food groups in dietary pattern analysis.

Food groups	Dietary Patterns Identified by principal component analysis	
	Healthy	Unhealthy
	(factor 1)	(factor 2)
Red meat	0.263	0.234
Processed meat	—	0.369
White fish and shellfish	—	0.224
Oily fish	0.375	—
Refined grains	—	0.302
Wholegrains	—	0.385
Potatoes	—	0.359
Chips	—	0.511
Crisps	—	0.554
Pizza	0.297	—
Low-fat dairy	0.305	—
High-fat dairy	—	0.466
Eggs	—	0.356
Dressings	0.29	0.382
Desserts	—	0.436
Chocolate	—	0.252
Sugars and sweets	—	0.415
Nuts	0.386	—
Soups	—	0.452
Alcohol	—	0.482
Fruit	0.517	—
Vegetables	0.558	0.243
Lutein-/zeaxanthin rich vegetables	0.714	—
Green leafy vegetables	0.696	—
Legumes	0.429	—
Alliums	0.59	—
Tomato	0.454	—
Refined breakfast cereal	—	0.256
Wholegrain breakfast cereal	0.274	—

Factor loadings under 0.2 were suppressed to highlight the main food groups in each factor.

Significant differences in age (p < 0.001) and BMI (p = 0.03) were observed across the fifths of the healthy dietary pattern (Table 1). Those who adhered least to the healthy diet (lowest fifth) were significantly older, whereas those who adhered most to the healthy dietary pattern (highest fifth) were significantly younger. There were no differences in the prevalence of hypertension, ischaemic heart disease, cerebrovascular accident, diabetes, alcohol status, or smoking status across fifths of the healthy dietary pattern and no differences were found in the prevalence of CKD stage 3–5, moderately reduced renal function, or severely reduced renal function across the categories.

For the unhealthy dietary pattern, significant differences across categories were observed for age (p = 0.01), smoking history (p = 0.03), alcohol use (p < 0.001), and diabetes mellitus (p = 0.007). No other differences were observed. The prevalence of CKD stage 3–5 differed across categories of the unhealthy dietary pattern with pairwise comparisons indicating fewer participants in the lowest fifth, i.e. less adherence to an unhealthy diet, with CKD stage 3–5 compared to all other categories (Table 3).

**Table 3:** Sample mean values and frequencies, for total sample and by fifths of adherence to the Unhealthy Dietary Pattern.

**Unhealthy Dietary Pattern**	Sample n = 1033	Q1 n = 207	Q2 n = 206	Q3 n = 206	Q4 n = 207	Q5 n = 207	*P*
Age, years (SD)	76.3 (8.0)	74.5 (7.6)^a^	76.5 (7.7)^b^	77.2 (8.1)^b^	77.0 (8.1)^b^	76.4 (8.2)^b^	0.01^†^
BMI, kg/m^2^ (SD)	24.6 (5.1)	24.5 (4.7)	24.5 (5.3)	24.2 (4.9)	24.9 (5.4)	24.7 (5.2)	0.75^†^
Hypertension, n (%)	566 (60.1)	111 (57.8)	116 (62.7)	120 (64.5)	104 (55.9)	115 (59.9)	0.43
Alcohol, n (%)	77 (8.2)	5 (2.6)	6 (3.2)	16 (8.6)*	18 (9.7)*	32 (16.7)*	<0.001
Ever smoked n (%)	35 (3.7)	3 (1.6)	3 (1.6)	8 (4.3)	13 (7.0)*	8 (4.2)	0.03
IHD, n (%)	107 (11.4)	24 (12.6)	24 (13.0)	16 (8.6)	24 (12.9)	19 (9.9)	0.56
CVA, n (%)	30 (3.2)	6 (3.1)	7 (3.8)	6 (3.2)	5 (2.7)	6 (3.1)	0.99
Diabetes mellitus [any] n (%)	31 (3.3)	5 (2.6)	14 (7.6)*	5 (2.7)	2 (1.1)	5 (2.6)	0.007
Type 2 diabetes	28 (3)	5 (2.6)	12 (6.5)	5 (2.7)	2 (1.1)	4 (2.1)	0.03
eGFR <60 mL/min/1.73 m^2^ n (%)	557 (60.6)	84 (45.9)	118 (64.8)*	108 (59.3)*	116 (63.4)*	131 (69.3)*	<0.001
eGFR <60 & ≥30 mL/min/1.73 m^2^ n (%)	534 (58.1)	81 (44.3)	111 (61.0)*	103 (56.6)*	112 (61.2)*	127 (67.2)*	<0.001
eGFR <30 mL/min/1.73 m^2^ n (%)	23 (2.5)	3 (1.6)	7 (3.8)	5 (2.7)	4 (2.2)	4 (2.1)	0.71

SD, standard deviation; IHD, Ischemic heart disease; CVA’ cerebrovascular accident; BMI, body mass index; eGFR, estimated glomerular filtration rate; Q1-5, fifths of dietary pattern adherence (Q1 lowest fifth, Q5 highest fifth). *Significant difference from lowest fifth in pairwise comparison for categorical variables via Mann-Whitney U test. ^†^P-value from ANOVA. ^a,b,c^ Where ANOVA P-value is significant, groups which are not significantly different from each other (by Student-Newman-Keuls) have been given the same subscript whereas groups which are different have been given different subscripts.

There was no evidence of a difference in the mean eGFR by category of healthy dietary pattern adherence before or after adjustment for covariates (Table 4). In contrast, there was evidence of a significant difference in the mean eGFR by adherence category to the unhealthy dietary pattern with an association of lower eGFR in Model 1 (unadjusted, p for trend < 0.001), Model 2 (adjusted for age and BMI p = 0.002), and Model 3 (adjusted for age, BMI, alcohol intake, smoking history, presence of diabetes, history of cerebrovascular accident, clinical diagnosis of hypertension, and history of ischemic heart disease, p < 0.001). Specifically those in the highest fifth of the unhealthy dietary pattern, i.e. greatest adherence, had significantly lower mean eGFR than those in the lowest fifth in the unadjusted model by an average 7 ml/min/1.73 m^2^ (60 ml/min/1.73 m^2^ for lowest fifth *vs* 53 ml/min/1.73 m^2^ for highest fifth, p < 0.001, Table 4) and this remained significant following adjustment for confounding variables (Models 2 and 3). Specifically, in the fully adjusted model, the difference in renal function between those in the highest and lowest fifths for the unhealthy dietary pattern was 6 ml/min/1.73 m^2^ (95% confidence interval [CI] −9, −3, p < 0.001, Table 4).

**Table 4:** Unadjusted and adjusted mean for healthy and unhealthy dietary patterns in participants of the Irish Nun Eye Study.

**Healthy**	Q1 (ref category)	Q2	Q3	Q4	Q5 (Most adherent)	P (p for trend)
	n = 206	n = 208	n = 206	n = 207	n = 206	
**Mean eGFR, (mL/min/1.73 m**^2^) **(SD)**						
Unadjusted	54 (17)	57 (17)	55 (15)	57 (16)	58 (16)	
**Mean difference in eGFR (in mL/min/1.73 m**^2^) **from reference category (95% CI):**						
Unadjusted	Ref.	+3.17 (0.18, 6.53)	+0.98 (−2.47, 4.44)	+2.68 (−0.68, 6.05)	+3.62 (0.25, 6.98)	0.17 (0.09)
Model 2	Ref.	+2.17 (−0.98, 5.32)	+0.02 (−3.25, 3.29)	+0.79 (−2.37, 3.96)	−0.37 (−3.60, 2.86)	0.51 (0.53)
Model 3	Ref.	+2.33 (−0.82, 5.47)	+0.21 (−3.04, 3.46)	+1.35 (−1.81, 4.51)	+0.01 (−3.22, 3.24)	0.49 (0.75)
**Unhealthy:**	n = 207	n = 206	n = 206	n = 207	n = 207	
**Mean eGFR, (mL/min/1.73 m2) (SD)**						
Unadjusted	60 (16)	56 (18)	57 (16)	55 (16)	53 (15)	
**Mean difference in eGFR (in mL/min/1.73 m**^2^) **from reference category (95% CI):**						
Unadjusted	Ref.	−4.08 (−7.41, −0.75)	−3.72 (−7.05, −0.38)	−5.08 (−8.41, −1.75)	−7.03 (−10.33, −3.73)	0.001 (<0.001)
Model 2	Ref.	−2.01 (−5.14, 1.11)	−1.38 (−4.51, 1.75)	−2.52 (−5.65, 0.61)	−5.22 (−8.32, −2.12)	0.02 (0.002)
Model 3	Ref.	−1.71 (−4.82, 1.41)	−1.57 (−4.70, 1.56)	−3.09 (−6.24, 0.05)	−5.68 (−8.81, −2.55)	0.007 (<0.001)

Ref., reference category; Q1-5, fifths of dietary pattern adherence (Q1 lowest fifth, Q5 highest fifth); SD, standard deviation; 95% CI, 95% confidence interval; eGFR, estimated glomerular filtration. Model 1 unadjusted; Model 2 adjusted for age and body mass index (BMI); Model 3 adjusted for age, BMI, presence of diabetes, presence of hypertension, ever smoking, presence/history of ischemic heart disease, presence/history of cerebrovascular accident and ever alcohol. CI; confidence interval.

In relation to CKD stage 3–5 risk, there was no significant trend for odds of CKD across the healthy dietary pattern adherence categories (Table 5). In contrast, greater adherence to an unhealthy dietary pattern was associated with greater risk of CKD stage 3–5 in both the unadjusted and adjusted models (fully adjusted odds ratio in the highest fifth compared with the lowest fifth: 2.62; 95% CI: 1.65, 4.15, p for trend <0.001).

**Table 5:** Odds ratios (95% confidence intervals) of chronic kidney disease across healthy and unhealthy dietary pattern.

Odds Ratio (95% CI)						
	Q1 (ref category)	Q2	Q3	Q4	Q5 (Most adherent)	P for trend
**Healthy**:	n = 206	n = 208	n = 206	n = 207	n = 206	
Unadjusted	Ref.	0.67 (0.44, 1.03)	0.98 (0.63, 1.53)	0.82 (0.53, 1.26)	0.63 (0.41, 0.97)	0.12
Model 2	Ref.	0.71 (0.45, 1.12)	1.07 (0.66, 1.73)	0.96 (0.61, 1.52)	0.90 (0.57, 1.44)	0.76
Model 3	Ref.	0.69 (0.43, 1.09)	1.04 (0.64, 1.69)	0.90 (0.56,1.43)	0.87 (0.54, 1.39)	0.97
**Unhealthy**:	n = 207	n = 206	n = 206	n = 207	n = 207	
Unadjusted	Ref.	2.17 (1.43, 3.31)	1.72 (1.14, 2.60)	2.04 (1.34, 3.10)	2.66 (1.74, 4.07)	<0.001
Model 2	Ref.	1.88 (1.21, 2.94)	1.39 (0.90, 2.16)	1.70 (1.09, 2.65)	2.38 (1.52, 3.73)	0.001
Model 3	Ref.	1.84 (1.17, 2.89)	1.44 (0.92, 2.25)	1.87 (1.19, 2.95)	2.62 (1.65, 4.15)	<0.001

Ref., reference category; 95% CI, 95% confidence interval; Model 1 unadjusted; Model 2 adjusted for age and body mass index (BMI); Model 3 adjusted for age, BMI, presence of diabetes, presence of hypertension, ever smoking, presence/history of ischemic heart disease, presence/history of cerebrovascular accident and ever alcohol.

## Discussion

We identified two dietary patterns in the current population which we named ‘healthy’ and ‘unhealthy’. There was a significant association between the unhealthy dietary pattern and both lower eGFR and increased odds of CKD stage 3–5. No significant relationship between adherence to a healthy dietary pattern and renal function was observed. Individuals in the category with the greatest adherence to the unhealthy dietary pattern had a mean eGFR that was 6 ml/min/1.73 m^2^ lower than the category whose diet least resembled the unhealthy dietary pattern. If causal, this difference is equivalent to the mean decline in renal function over a period of approximately 14 years based on the renal decline of older adults in the Cardiovascular Health Study^23^.

The unhealthy dietary pattern identified in the current study bore similarities to unhealthy “Western” type dietary patterns used in other studies which similarly included greater consumption of red and/or processed meat and refined grains, increased consumption of sweets, desserts, high-fat dairy, sugar, fast foods such as chips, and low intakes of fruits and vegetables^2,24,25^. The unhealthy dietary pattern components/characteristics identified in this study support the external validity of our findings with comparisons to other populations consuming similar unhealthy and Western-style diets.

Western dietary patterns have previously been associated with type 2 diabetes^24^ and have been implicated in the development of hypertension^25^. Hypertension and diabetes are the leading causes of CKD worldwide^26^ and offer hypothetical mechanisms for the negative effects associated with Western diets. In our data, no significant associations were found between hypertension and unhealthy dietary pattern, while only 3% of our sample had type 2 diabetes. Furthermore, as with other studies examining the association between dietary patterns and renal function^5–11^, we controlled for diabetes and hypertension in our analyses and the associations observed between the unhealthy dietary pattern and poorer renal function remained significant after adjustment, suggesting independence from hypertension and diabetes.

Similarly, both smoking^27^ and alcohol^28^ consumption influence CKD risk and although the prevalence of both was low in this population, they were more commonly found in those with an unhealthy dietary pattern. Nevertheless, the associations reported remained significant following adjustment for these potential confounding variables. High BMI has been previously associated with CKD^29^ and with unhealthy dietary patterns^30,31^. In our sample, BMI was not associated with an unhealthy dietary pattern and the associations between the unhealthy dietary pattern and renal function remained significant in models adjusting for BMI. BMI, therefore, does not appear to explain the relationship observed in this instance and for which the mechanism remains to be determined.

The healthy dietary pattern we identified had considerable overlap with the Mediterranean and DASH diets, as well as various national dietary guidelines. Both Mediterranean and DASH dietary patterns are characterized by comparatively high intakes of vegetables, fish, whole grains, and low consumption of red and processed meats, sugar-sweetened, high-fat, and high-sodium foods compared to modernized Western diets^25^. Cross-sectional studies have previously reported that adherence to a Mediterranean diet is associated with higher creatinine clearance^8^, while adherence to a DASH diet is associated with reduced risk of CKD^6^. Similarly, a cross-sectional association between eGFR <60 ml/min/1.73 m^2^ and poorer adherence to a modified Healthy Eating Index for Australia, that had considerable overlap with both the DASH and Mediterranean diets, has been reported^32^. Longitudinal studies also support the association between such healthy dietary patterns and improved renal function. Mediterranean dietary pattern studies with up to 10 years follow-up showed lower risk of CKD^9,10^, while the DASH diet has been associated with both slowing the rate of eGFR decline by >30% over 11 years^5^ and reduced CKD risk over 23 years of follow-up^7^. Based on this evidence and the similarity of foods present within the *a-priori* and *a-posteriori* defined dietary patterns, it is unclear why we failed to observe an association between the healthy dietary pattern and renal function^6^. A larger sample size may have reduced the risk of a false negative finding. Previously, Lin *et al*. (2011) reported significant associations between the DASH diet and eGFR but similarly found no association between an *a-posteriori* derived healthy eating pattern and eGFR in the same study^5^. They suggest that the weightings given to food types in their *a-posteriori* derived dietary patterns do not accurately represent those food groups that are most important for eGFR decline compared to those for the DASH diet^5^.

This study had several strengths. This study provides novel evidence of the association between dietary patterns and CKD in a large European population, and data collection was standardized and collected for a wide range of potential confounding variables. The uniformity of lifestyles within the convent setting of this study minimized the risk of potential confounding. We used a culturally appropriate FFQ, designed for use in British and Irish populations. The FFQ comprised a large number of food items (n = 170), which improved the accuracy of dietary data collection and reduced the risk of under-reporting of consumption of local foods. The foods consumed by this population are thought to more closely and consistently reflect traditional regional diets.

Examining dietary patterns rather than isolated nutrients or foods confers benefits in comparison to studies which examined individual nutrients, the effects of which are prone to interaction with other dietary factors^33,34^. Conversely, this approach does not account for the effects of individual nutrients of interest. The use of *a-posteriori* dietary patterns derived specifically from the study sample may also increase the applicability of the findings to the context and culture of regional populations. Furthermore, the *a-posteriori* approach used to identify the dietary patterns was not based on predetermined hypotheses linking diet and health outcomes.

Limitations of the study include its cross-sectional design which prevents causal inference and measurement of associations between dietary patterns and CKD over time. In addition, any effect of CKD diagnosis on dietary intake likewise could not be assessed. A longitudinal study assessing CKD incidence would best address these weaknesses.

The study setting may limit the generalizability of our findings. The population was all female and dietary associations with CKD may differ in men. The cloistered and highly homogenous lifestyle of the study population may also limit the applicability of our findings to the general population. Furthermore, the regional food choices and use of a region-specific FFQ, also limits the generalisability of the study to other regions.

Renal function was estimated using the CKD-EPI equation which provides better accuracy than estimation via the earlier “MDRD” equation, particularly for eGFR values >60 ml/min/1.73 m^2^ ^35^. CKD was categorized on the basis of a single serum creatinine measurement using a renal function cut-off value of 60 ml/min/1.73 m^2^. This differs from the clinical definition of CKD which assumes an eGFR <60 ml/min/1.73 m^2^ for 3 months or more in the absence of persistent albuminuria, or any eGFR in the presence of persistent albuminuria of ≥3 mg/mmol. Therefore, the strength of the associations between dietary patterns and individuals defined as having CKD in this study may not necessarily reflect associations with clinically diagnosed CKD in general.

Inherent error is common with FFQs, particularly in relation to the accuracy of portion size and frequency of consumption^36^. As a result, FFQs are considered to be a more accurate tool for assessing the types of food consumed rather than the quantity of food consumed. However, the regimented lifestyles of the participants are likely to have minimized dietary variability. While energy intake was not measured as part of the dietary analysis, the risk of misreporting portion sizes in the FFQ was minimized by the use of visual aids and standardized photographs of food portions of commonly consumed foods. Use of a regionally appropriate FFQ helped reduce potential for under-reporting and misreporting of dietary intake.

The creation of food groups for inclusion in principal component analysis relies on informed decision making by research staff and may not have reflected food groupings most pertinent to CKD. The dietary patterns used in this study accounted for only 16% of dietary variance. This variance is consistent with studies conducted in other Western populations but highlights that relevant unidentified dietary patterns may exist. Dietary patterns are intimately linked to lifestyle and socioeconomic contexts and there may have been important confounding variables we failed to consider within our model. Our study sample was drawn from a population with relatively homogenous socioeconomic status and this is therefore unlikely to be an important confounder. Furthermore, the ranges of factor scores for the healthy and unhealthy dietary pattern were similar, indicating affordability of a healthy dietary pattern did not explain the lack of association.

A further limitation of dietary pattern analysis is that while this method provides an indication of the type of foods/food groups consumed within a specific dietary pattern, it does not provide any information on the amount of each food consumed.

The results of this study provide support for the role of diet in CKD. In this population, an unhealthy dietary pattern was independently associated with lower eGFR and an increased risk of CKD. The lack of association of renal function with a healthy dietary pattern suggests that inclusion of healthy foods in the diet may not necessarily afford protection from renal decline but rather highlights the potential detrimental effects exerted by unhealthy dietary patterns, suggesting minimizing the intake of certain foods may be required. Further longitudinal research is necessary in order to establish whether change in overall dietary intake over time can ameliorate or exacerbate CKD risk and eGFR decline.

## Methods

### Study design and population

The current study used data collected as part of the Irish Nun Eye Study (INES) study, a cross-sectional study which primarily examined the association between age-related macular degeneration (AMD) and light exposure in Irish nuns, a well characterized and homogenous population. Participants were recruited from 126 convents across Ireland between 2007 and 2009. For inclusion, participants had to be of Irish descent, white ethnicity, aged ≥55 years, and resident in a convent for 25 years or more. This research was conducted in line with the Declaration of Helsinki. The Office for Research Ethics Committee Northern Ireland granted ethical approval prior to the commencement of research and informed written consent was obtained from all participants. The study design and sampling procedures have been previously described^37–39^.

### Variables and data collection

All measurements were carried out within the convents by trained researchers. Food intake was estimated using the validated semi-quantitative FFQ of the Scottish Collaborative Group^40^. Participants recorded the frequency of consumption of 170 foods of specified portion sizes during the 2–3 months preceding the completion of the questionnaire. Food portion size photographs were also used to aid portion size estimation. Only complete FFQs were included for analysis. FFQ data were subsequently converted into grams using the standardized Food Portion Sizes^41^.

Blood samples were obtained from participants and frozen at −80 °C until analysis. Renal function was measured as eGFR calculated from serum creatinine values using the CKD-EPI equation^35^. Those with eGFR <60 mL/min/1.73 m^2^ were classified as having CKD. Renal function was further divided into moderately reduced renal function, eGFR <60 mL/min/1.73 m^2^ but greater than 30 mL/min/1.73 m^2^, and severely reduced renal function, eGFR <30 mL/min/1.73 m^2^.

A structured interview was conducted to obtain lifestyle and demographic data, including age, alcohol intake, smoking history, and self-reported disease status. Height and weight were measured and BMI calculated as weight (kg)/height (m^2^).

### Statistical analyses

Statistical analyses were conducted on IBM SPSS v24 (Chicago, Illinois, USA). Continuous variables were summarized using means and standard deviations, all continuous variables were normally distributed. Categorical variables were summarized using frequencies and percentages. The dietary pattern analysis has previously been described by Neville *et al*.^39^. In brief, the food items present in the FFQ were manually grouped into 38 food groups based on macronutrient content and food type (Supplementary Table S1). Dietary patterns were generated from the food groups using principal component analysis with Varimax rotation^42,43^.

Factors above the breakpoint on the scree plot were retained. Food groups with factor loadings greater than 0.2 were included in further analysis. Weighted factor scores were computed for each participant by summing the intakes of food groups and weighting by their factor loading. Dietary pattern scores generated from principle component analysis were analyzed according to fifths of adherence to the dietary pattern, with the lowest fifth (reference category) reflecting lowest adherence to the dietary pattern and highest fifth reflecting highest adherence to the dietary pattern.

One way analysis of variance (ANOVA) was used to test for differences in means between fifths for continuous variables. Student-Neuman-Keuls (SNK) *post-hoc* test was used for comparison between fifths controlling for multiple comparisons. Kruskal-Wallis test was used to examine differences between fifths for categorical variables. *Post-hoc* pairwise comparisons were conducted using Mann-Whitney U tests to determine the location of any differences.

Multivariable linear regression was used to calculate the difference in mean eGFR (and 95% CIs) between fifths of dietary pattern adherence and to calculate differences in means adjusted for covariates described below. Logistic regression was used to calculate odds ratios (and 95% CIs) for the association between dietary pattern adherence and risk of CKD (based upon eGFR <60 mL/min/1.73 m^2^) and calculate ORs adjusted for the covariates described below. For multivariable linear and logistic regression analyses three models were used: Model 1: unadjusted model; Model 2: adjusted for age and BMI and Model 3: adjusted for age, BMI, alcohol intake, smoking history, presence of diabetes, history of cerebrovascular accident, clinical diagnosis of hypertension, and history of ischemic heart disease. Variables included within adjusted models were known associated CKD risk factors.

The datasets generated during and/or analysed during the current study are available from the corresponding author on reasonable request.

## Electronic supplementary material

Supplementary table S1. Food items included in food groups for dietary pattern analysis
