# Supplementary material for: Dietary patterns and chronic kidney disease: a cross-sectional association in the Irish Nun Eye Study
Source: Sci Rep. 2018 Apr 27;8:6654. doi: 10.1038/s41598-018-25067-7 (PMC5923202; doi:10.1038/s41598-018-25067-7)
Supplement: Supplementary file 1 — Supplementary table S1. Food items included in food groups for dietary pattern analysis [file 41598_2018_25067_MOESM1_ESM.docx]

**Supplementary Material**

Title:

Dietary patterns and chronic kidney disease: a cross-sectional association in the Irish Nun Eye Study

Authors: *Euan N Paterson BSc,^a^ *Charlotte E Neville PhD,^a^ Giuliana Silvestri MD,^b^ Shannon Montgomery BSc,^a^ Evelyn Moore RGN,^b^ Vittorio Silvestri HND,^b^ Christopher C Cardwell PhD,^a^ Tom J MacGillivray PhD,^c^ Alexander P Maxwell MD,^a^ Jayne V Woodside PhD,^a,d^ Gareth J McKay PhD^a^

*Both authors contributed equally to this work.

^a^Centre for Public Health, Queen’s University Belfast, Belfast, Northern Ireland

^b^Department of Ophthalmology, Belfast Health and Social Care Trust, Royal Hospital, Belfast, Northern Ireland

^c^Centre for Clinical Brain Sciences, The University of Edinburgh, Edinburgh, Scotland

^d^UKCRC Centre of Excellence for Public Health, School of Medicine, Dentistry and Biomedical Sciences, Queen’s University Belfast, Belfast, BT12 6BJ, United Kingdom

Corresponding author:

Dr Gareth J McKay Ph.D.

Centre for Public Health,

Queen’s University Belfast,

Institute of Clinical Sciences, Block B,

Royal Victoria Hospital,

Belfast

BT12 6BA

+44 (0)28 9097 8958

[g.j.mckay@qub.ac.uk](mailto:g.j.mckay@qub.ac.uk)

**Supplementary table S1**. **Food items included in food groups for dietary pattern analysis**

| **Food groups** | **Food items included (from the FFQ)** |
| --- | --- |
| Red meat | Minced beef, beef-burgers (fried), roast beef, roast pork |
| Organ meat | Liver |
| Poultry | Roast chicken |
| Processed meat | Grilled pork sausages, bacon gammon joint, black pudding, ham, salami, chicken pie |
| White fish and shellfish | Fish fingers, battered cod, poached white fish (cod), smoked white fish (cod), fish cakes, prawns, mussels |
| Oily fish | Fried mackerel, grilled salmon, smoked salmon, tinned tuna, sardines |
| Refined grains (excludes refined cereals) | White sliced bread, soft white roll, garlic bread, pitta bread, white rice, pasta (spaghetti), noodles, scones, crackers, savoury pancakes |
| Wholegrains (excludes wholegrain cereals) | Brown rice, oatcakes |
| Potatoes without waffles and roasts | Boiled potatoes, mashed potatoes, potato salad |
| Chips with waffles and roasts | Home-cooked and retail chips, potato waffles, roast potatoes |
| Crisps | Crisps, reduced fat crisps, tortilla crisps |
| Pizza | Pizza |
| Low fat dairy excluding Horlicks | Semi-skimmed milk, skimmed milk, low fat yogurt, low calorie yogurt, low fat cheese, cottage cheese |
| High fat dairy | Full fat milk, full fat yogurt, fromage frais, cream, cheddar cheese, cheese spread, Philadelphia cheese, dried milk |
| Eggs | Boiled hen's egg, fried egg, scrambled egg, quiche |
| Dressings/sauces/condiments | White sauce, ketchup, mayonnaise, oil and vinegar dressing, tomato chutney, gravy, marmite |
| Desserts (excluding biscuits) | Milk-based pudding, sponge puddings, cheesecake, fruit pie, mousse, custard, Cornetto, other icecream, sponge cake, sponge cake with jam/cream/icing, fruit cake, doughnuts |
| Chocolate excluding hot chocolate | Dairy milk bar |
| Biscuits | Cereal bars, digestive biscuits, custard creams, shortbread, chocolate coated biscuits |
| Sugar and sweets | Toffees, boiled sweets, fruit gums, jam |
| Nuts | Peanuts, unsalted nuts, peanut butter |
| Soup | Home-made soup (vegetable), tinned soup (tomato), instant soup |
| Tea | Tea, herbal tea |
| Coffee | Instant coffee, decaffeinated coffee, cappuccino |
| Alcohol | Low alcohol beer, dark beer (stout), light beer (lager), white wine, red wine, sherry/port, spirits/liqueurs, alcopops, cider |
| Fruit juice | Pure orange juice, tomato juice |
| Fruit | Fresh fruit salad, tinned fruit, apples, bananas, oranges, pears, peaches, kiwi, mixed dried fruit, grapes |
| Vegetables | Mixed vegetable stir-fry, tinned vegetables, coleslaw, sweet peppers |
| Lutein-zeaxanthin rich vegetables | Carrots, sweetcorn, lettuce |
| Cruciferous green leafy vegetables | Cabbage, brussel sprouts, broccoli, spinach, cauliflower |
| Legumes | Peas, baked beans, soya beans, kidney beans, lentils |
| Allium family | Leeks, onions |
| Tomato group | Tomatoes, tomato pasta sauce |
| Meat replacement products/vegetarian | Soya milk, vegeburgers, quorn, tofu |
| Soft drinks | Blackcurrant squash, fruit squash, diet cola, regular fizzy drinks |
| Hot chocolate and Horlicks | Hot chocolate, Horlicks |
| Refined breakfast cereals | Cornflakes, Crunchy nut cornflakes |
| Wholegrain breakfast cereals | Porridge, all bran, muesli, weetabix |
